# Organerhaltende Behandlung bei nodal-positiven Harnblasenkarzinomen

**DOI:** 10.1007/s00066-023-02156-9

**Published:** 2023-09-29

**Authors:** Małgorzata Toroń, Antoni Wołoszyn

**Affiliations:** 1 Klinik für Strahlentherapie, Christian-Albrechts-Universität zu Kiel, Kiel, Deutschland; 2 Schlesische Medizinische Universität in Katowice, Katowice, Polen

## Originalarbeit

Swinton M, Mariam NBG, Tan JL et al (2023) Bladder-Sparing Treatment With Radical Dose Radiotherapy Is an Effective Alternative to Radical Cystectomy in Patients With Clinically Node-Positive Nonmetastatic Bladder Cancer. J Clin Oncol. 2023 Jul 21: JCO2300725. 10.1200/JCO.23.00725.

## Hintergrund

Die organerhaltende Behandlung mit simultaner Radiochemotherapie (in den meisten Publikationen als trimodale Therapie [TMT] bezeichnet) ist eine äquieffektive Alternative zur radikalen Zystektomie; diese Tatsache ist zwar nicht durch randomisierte Studien mit einem direkten Vergleich beider Verfahren bestätigt, aber die beste verfügbare Evidenz aus prospektiven Studien und Kohortenanalysen in unterschiedlichen Ländern belegt die Gleichwertigkeit einer Organerhaltung im Hinblick auf das Langzeitüberleben [1–3]. Eine aktuelle Kohortenanalyse fand sogar einen signifikanten Überlebensvorteil für die organerhaltende trimodale Therapie [8]. Dennoch wird in den Leitlinien urologischer Fachgesellschaften weiterhin die radikale Zystektomie als Verfahren der ersten Wahl und Goldstandard dargestellt [6, 7]. Es ist aus radioonkologischer Sicht berechtigt und dringend notwendig, das zu hinterfragen [4].

Die meisten Patienten, die wegen eines muskelinvasiven Blasenkarzinoms für eine Radiochemotherapie infrage kommen, sind in der Bildgebung nodal-negativ. Für die Subgruppe mit klinisch detektierten regionalen Lymphknotenmetastasen gibt es kaum Daten, da klinischer LK-Befall in den meisten Studien nicht als einzelner Prognosefaktor untersucht wurde. Eine aktuelle britische Analyse bestätigt, dass auch in diesem Kollektiv die Radio(chemo)therapie eine äquieffektive und sehr sinnvolle Alternative zur Zystektomie ist [5].

## Material und Methoden

Für die Analyse wurden die Daten von Patienten mit Harnblasenkarzinomen der Kategorie cN1 M0 ausgewertet, die zwischen Juli 2012 und März 2021 an vier britischen Onkologiezentren behandelt worden waren. Bezüglich der Behandlungsverfahren wurde zwischen kurativ intendierter und palliativer Therapie unterschieden; kurative Therapie bedeutete radikale Zystektomie oder „radikale“ Radiotherapie, jeweils allein oder in Kombination mit Chemotherapie. Palliative Therapieverfahren waren palliative Radiotherapie oder Chemotherapie oder „best supportive care“. Ausgewertet wurden das Gesamtüberleben (OS) und das progressionsfreie Überleben (PFS).

## Ergebnisse

Die Untersuchung umfasste eine Kohorte von 287 Patienten mit cN1-M0-Urothelkarzinomen der Harnblase. Das mittlere mediane Gesamtüberleben (OS) betrug 1,55 Jahre, wobei 39 % der Patienten mindestens 2 Jahre überlebten und nur 19 % 5 Jahre überlebten. Das mediane progressionsfreie Überleben (PFS) betrug 0,95 Jahre mit einer Progressionsfreiheit von 28 % nach 2 und 17 % nach 5 Jahren.

163 Patienten hatten eine kurativ intendierte Therapie erhalten, davon 76 eine Zystektomie und 87 eine Radiotherapie; die 2‑Jahres-Überlebensrate betrug in diesem Kollektiv 56 % (die 5‑Jahres-Überlebensrate wird im Text der Arbeit nicht angegeben, betrug nach den Überlebenskurven aber etwa 25 %). Bei den 124 Patienten mit palliativer Therapie betrug die 2‑Jahres-Überlebensrate 18 %; diese palliativen Patienten waren signifikant älter (im Median 73,5 vs. 69 Jahre) und hatten höhere T‑ und N‑Kategorien und einen schlechteren Allgemeinzustand als die kurativen Patienten.

Bei den kurativ behandelten Patienten zeigte sich kein Unterschied zwischen Radiotherapie und Zystektomie, weder im Gesamtüberleben (*p* = 0,5) noch im progressionsfreien Überleben (*p* = 0,07; die absoluten Zahlen waren für die Radiotherapie günstiger). Das galt sowohl bei Op. oder RT als Monotherapie als auch bei Kombination mit Chemotherapie; Patienten, die eine Chemotherapie erhalten hatten, zeigten etwas längere Überlebenszeiten, aber der Effekt war nicht signifikant. Bei den mit kurativer Intention bestrahlten Patienten war in 59 Fällen nur die Blase bestrahlt worden und in 27 Fällen auch die regionalen Lymphknoten; die zusätzliche LK-Bestrahlung war mit einem tendenziell schlechteren Überleben (aber nicht signifikant) verbunden. In multivariater Analyse war bei den kurativ behandelten Patienten ein höheres klinisches N‑Stadium (cN2/3 vs. cN1) ein signifikanter Risikofaktor für ein schlechteres Gesamtüberleben (HR = 1,72; *p* = 0,007) und progressionsfreies Überleben (HR = 1,82; *p* = 0,007); Alter, Allgemeinzustand und T‑Kategorie waren nicht signifikant.

## Schlussfolgerung der Autoren

Auch bei Patienten mit klinisch positivem Lymphknotenbefall und nichtmetastasiertem Blasenkarzinom (cN1 M0) ist eine organerhaltende Behandlung eine äquieffektive Alternative zur Zystektomie.

## Kommentar

Diese retrospektive Studie bestätigt, was vermutet werden konnte, aber so eindeutig bisher nicht belegt war: Auch bei nodal-positiven Patienten sollte man eine Radiochemotherapie als Option anbieten und empfehlen.

Diese Studie bietet die beste Evidenz für dieses eher seltene Patientenkollektiv, aber sie hat wie alle retrospektiven Analysen einige Schwächen:

1. Ein relativ hoher Anteil von Patienten (immerhin 43 %) erhielt trotz der noch lokoregional begrenzten Erkrankung eine palliative Therapie, und das war niemals eine Zystektomie. Dieser palliative Anteil erscheint für deutsche Verhältnisse sehr hoch, und die Selektionskriterien sind unklar. Operateure könnten das als Ausdruck eines gewissen therapeutischen Nihilismus in einem maximal belasteten Gesundheitssystem interpretieren. Vielleicht sind unsere britischen Kollegen aber einfach auch realitätsbewusster und entscheiden sich bei schlechter Prognose eher für eine schonende Therapie im Interesse der Patienten.
2. Für die in kurativer Intention behandelten nodal-positiven Patienten wurden akzeptable und im internationalen Vergleich sehr gute Langzeitüberlebensraten erreicht. Das bestätigt, dass eine solche Therapie bei entsprechend selektionierten Patienten sinnvoll ist.
3. Der wichtigste prognostische Faktor war das Ausmaß des Lymphknotenbefalls. Das sollte man bei Therapieentscheidungen berücksichtigen.
4. Dass die Bestrahlung nur der Blase mit einem besseren Outcome verbunden war als die zusätzliche Bestrahlung der Lymphknoten, erscheint diskrepant. Wenn man allerdings unterstellt, dass ein makroskopischer Tumor immer behandelt (bestrahlt) wurde, ist das durchaus plausibel. Wenn eine Bestrahlung nur der Blase alle befallenen (direkt paravesikalen) Lymphknoten erfasst und die Erweiterung des Zielvolumens zur Mitbestrahlung der Lymphknoten als Ausdruck eines ausgedehnteren LK-Befalls anzusehen ist, wären die Ergebnisse gut erklärbar. Indirekt würde das aber auch bedeuten, dass eine prophylaktische Bestrahlung der nicht befallenen Lymphknoten vermutlich keinen relevanten Stellenwert hat.

### Fazit

Eine Radiochemotherapie oder Radiotherapie ist immer die beste Option beim muskelinvasiven Harnblasenkarzinom. Das betrifft auch nodal-positive Patienten, und es gilt für kurative und palliative Therapieintentionen.

*Małgorzata Toroń, Antoni Wołoszyn, Kiel/Katowice*
